# Anti-IL-20 monoclonal antibody inhibited tumor growth in hepatocellular carcinoma

**DOI:** 10.1038/s41598-017-17054-1

**Published:** 2017-12-14

**Authors:** Yi-Shu Chiu, Chung-Hsi Hsing, Chien-Feng Li, Chon-Yee Lee, Yu-Hsiang Hsu, Ming-Shi Chang

**Affiliations:** 1 Institute of Biopharmaceutical Sciences, College of Medicine, National Cheng Kung University, Tainan, Taiwan; 2 Department of Biochemistry and Molecular Biology, College of Medicine, National Cheng Kung University, Tainan, Taiwan; 3 Department of Anesthesiology, Chi-Mei Medical Center, Tainan, Taiwan; 4 Department of Pathology, Chi-Mei Medical Center, Tainan, Taiwan; 5 National Institute of Cancer Research, National Health Research Institutes, Tainan, Taiwan; 6 Department of Biotechnology, Southern Taiwan University of Science and Technology, Tainan, Taiwan; 7 Institute of Clinical Medicine, College of Medicine, National Cheng Kung University, Tainan, Taiwan; 8 Research Center of Clinical Medicine, National Cheng Kung University Hospital, College of Medicine, National Cheng Kung University, Tainan, Taiwan

## Abstract

Interleukin (IL)-20 is a proinflammatory cytokine involved in rheumatoid arthritis, atherosclerosis, and osteoporosis. However, the role of IL-20 in hepatocellular carcinoma (HCC) is unclear. We explored the function of IL-20 in HCC. Tumor tissue samples were analyzed the expression of IL-20 and cyclin D1 by using immunohistochemistry staining and quantitative real-time polymerase chain reaction (qRT-PCR) analysis. To examine the role of anti-IL-20 monoclonal antibody (7E) in tumor growth, BALB/c mice was injected with ML-1 cells and treated with 7E. HCC tumor tissue expressed higher levels of IL-20 than did non-tumor tissue. High IL-20 expression in HCC was correlated with poor overall survival (relative risk:>3). IL-20 and cyclin D1 expression were also highly correlated in HCC patient specimens and 3 human HCC cell lines. IL-20 also increased cell proliferation and migration, and it regulated matrix metalloproteinase (MMP)-13, tumor necrosis factor (TNF)-α, cyclin D1, and p21^WAF1^ expression in ML-1 cells. 7E attenuated tumor growth in mice inoculated with ML-1 cells. The expression of cyclin D1, TNF-α, MMP-9, and vascular endothelial growth factor was significantly inhibited after 7E treatment. The findings of this study suggest that IL-20 plays a role in the tumor progression of HCC.

## Introduction

Hepatocellular carcinoma (HCC) is one of the most common human cancers and a leading cause of cancer-related deaths worldwide. HCC has a multiple-step progression with a high evidence of association with chronic inflammation exposure induced by environmental toxin intake and chronic viral infection, such as HBV or HCV^1^. The regulatory mechanism of chronic inflammation leading to the formation of HCC tumors is unclear. Recent investigation has demonstrated the contributions of inflammation to the HCC progression and link these processes to overall HCC development^2–4^.

Cyclin D1 is one of the major regulators of the cell cycle and is a marker for human malignancies. The growth-promoting functions and deregulated expression of cyclin D1 increases tumor proliferation in several human cancers. The cyclin D1/CDK complex regulates the transition from the G1 phase to the S phase. The activities of these cyclin D1/CDK complexes are negatively regulated by the CDK inhibitor p21^WAF1^. Cyclin D1 is also frequently overexpressed in a variety of human carcinomas, including HCC. Cyclin D1 overexpression may be important in the development of human HCC^5^.

Interleukin (IL)-20 is a member of the IL-10 family, which includes IL-10, -19, -20, -22, -24, and -26. IL-20 signals through two types of receptor complex: IL-20R1/IL-20R2 and IL-22R1/IL-20R2^6^. IL-20 targets keratinocytes, endothelial cells, synovial fibroblasts, and several types of tumor cells, especially squamous cell carcinoma of the skin, tongue, esophagus, and lung^7^. IL-20 is involved in inflammation, angiogenesis, arteriogenesis, and chemotaxis, all of which are important for the pathogenesis of psoriasis, atherosclerosis, rheumatoid arthritis, and ischemic disorders^7–9^.

IL-20 is well known as an inflammatory cytokine involved in many immune diseases and tumorigenesis. We recently reported^10^ that IL-20 promotes tumor progression and affects clinical outcome in breast cancer. We also found that anti-IL-20 monoclonal antibody (mAb) 7E alleviates inflammation in oral cancer and suppresses tumor growth^11^. The role of IL-20 in HCC is still unclear. In this study, we investigated whether IL-20 is involved in the pathogenesis of HCC and analyzed the mechanism of IL-20 in HCC. We also explored whether 7E inhibits tumor growth in a murine model of HCC.

## Results

### IL-20 expression in tumor tissue was correlated with clinical outcome

**Figure 1:**
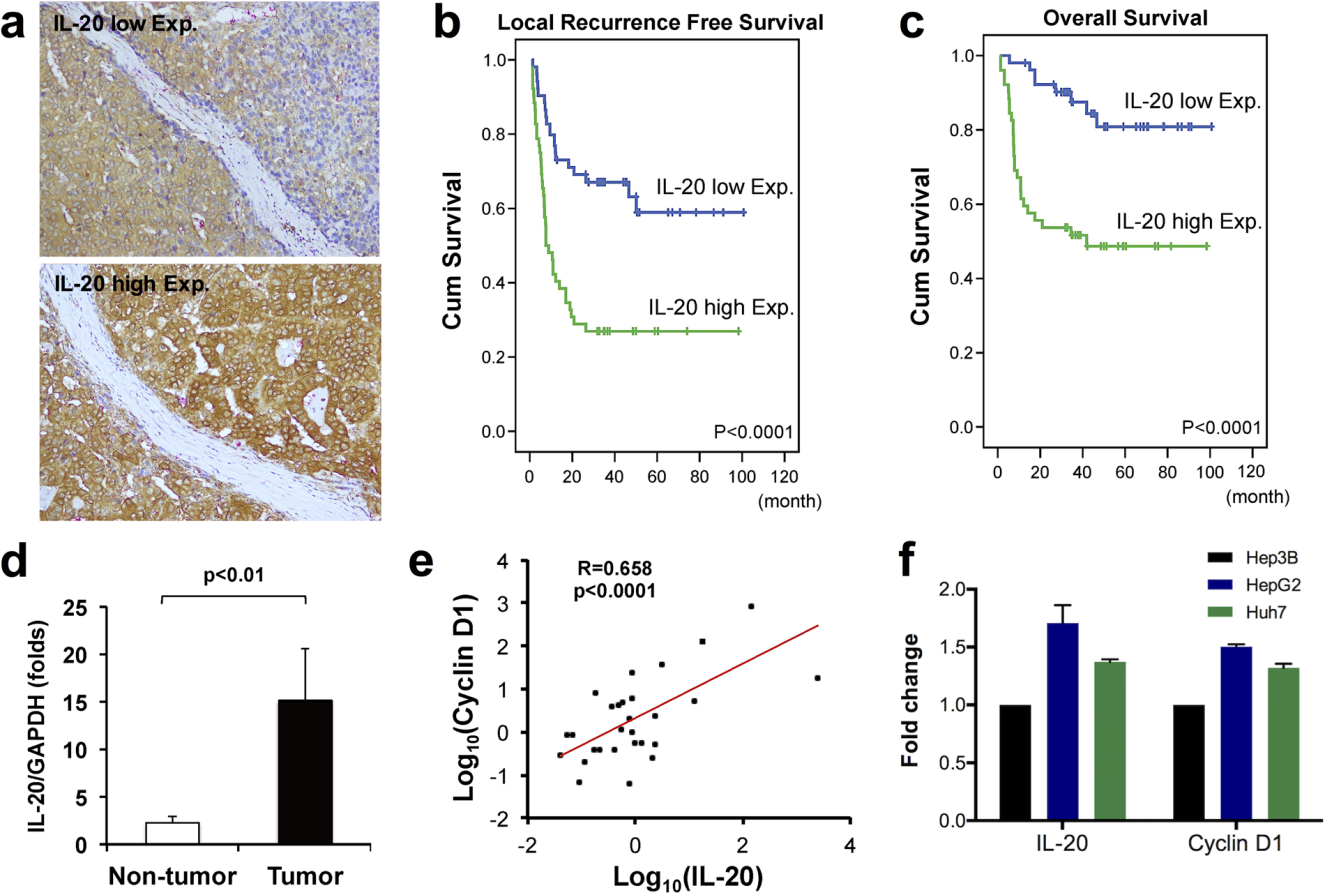
IL-20 expression in HCC tumors was correlated with overall survival, local recurrence free survival, and cyclin D1 expression. (**a**) Expression of IL-20 in 104 HCC tissues were determined using IHC staining and classified as IL-20 low expression (IL-20 low Exp.) and high IL-20 high expression (IL-20 high Exp.) (400X). The expression levels of IL-20 in tissue samples were analyzed using H scoring and graded as low IL-20 expression (IL-20-L, H < medium, n = 52) and high IL-20 expression (IL-20-H, H > medium, n = 52). Kaplan-Meier plots were used to predict (**b**) local recurrence free survival and (**c**) overall survival based on IL-20 expression levels. (**d**) The mRNA expression of IL-20 in non-tumor tissue and tumor tissue. Quantification analysis of mRNA was normalized with GAPDH. Values are means ± SD. Data are representative of three independent experiments. (**e**) The correlation of IL-20 and cyclin D1 mRNA expression in 26 patients with HCC. Values represent log10 of mRNA expression change in each individual patient. Each point represents each individual. (**f**) IL-20 and cyclin D1 mRNA expression in human hepatoma cell lines HepG2, Hep3B, and Huh7 were analyzed using qRT-PCR with specific primers.

One hundred and four HCC samples were immunohistochemistry stained with 7E and yielded 52 cases of high and 52 cases of low IL-20 expression (Fig. 1a). Higher IL-20 expression was significantly related to the Child-Pugh Classification (*P* = 0.010), tumor multiplicity (*P* = 0.003), tumor differentiation (*P* = 0.003), the primary tumor (*P* = 0.002), the American Joint Committee on Cancer TNM system stage (*P* = 0.002), the CLIP score (*P* = 0.001), and the Okuda stage (*P* = 0.003) (Table 1). In addition, univariate survival analysis showed that IL-20 expression was highly correlated with poor local recurrence-free survival (*P* < 0.0001, Fig. 1b) and poor overall survival (*P* < 0.0001, Fig. 1c) (Table 2). In multivariate survival analysis, high IL-20 expression was also correlated with poor overall survival (RR = 3.025, 95% CI = 1.110–8.241, *P* = 0.030) and poor local recurrence-free survival (RR = 2.348, 95% CI = 1.211–4.553, *P* = 0.011) (Table 3).

**Table 1:** Correlations between IL-20 expression to various clinicopathological parameters.

	Number	IL-20 Exp.		P-value
		Low	High	
**Sex**				0.135
Male	84	39	45	
Female	20	13	7	
**Age** (**years**)				1.000
<60	48	24	24	
≧60	56	28	28	
**Hepatitis**				0.529
HBV	54	29	25	
HCV	38	17	21	
Both	4	1	3	
None	8	5	3	
**Pugh-Child’s Classification**				**0**.010*
A	85	45	40	
B	6	0	6	
C	3	0	3	
**AFP**				0.292
<400	75	42	33	
≧400	23	10	13	
**Tumor Multiplicity**				**0**.003*
Solitary	61	38	23	
Multiple	43	14	29	
**Differentiation**				**0**.003*
Well/Moderately differentiated	79	46	33	
Poorly differentiated	25	6	19	
**Primary Tumor** (**pT**)				**0**.002*
pT1	42	27	15	
pT2	32	18	14	
pT3–4	30	7	23	
**AJCC Stage**				**0**.002*
Stage I	40	26	14	
Stage II	32	18	14	
Stage III-V	32	8	24	
**CLIP score**				**0**.001*
0–1	63	37	26	
2–3	30	7	23	
**Okuda stage**				**0**.003*
I	62	36	26	
II-III	31	8	23	

HBV: viral hepatitis B.

HCV: viral hepatitis C.

AFP: alpha fetal protein OS: overall survival.

LRFS: local recurrence free survival.

CLIP: The Cancer of the Liver Italian Program.

AJCC: The American Joint Committee on Cancer TNM system.

**Table 2:** Univariate survival analyses.

Parameter	No. of Case	OS		LRFS	
		No. Event	P-value	No. Event	P-value
**Sex**			0.8128		
Male	84	27		47	0.3931
Female	20	8		10	
**Age (years)**			0.6115		0.1427
<60	48	15		22	
≧60	56	20		35	
**Pugh-Child’s Classification**					
A	85	23	**<0**.0001*	43	**<0**.0001*
B	6	6		6	
C	3	3		3	
AFP			0.0556		0.1840
<400	75	19		37	
≧400	23	10		14	
**Tumor Multiplicity**			**<0**.0001*		**<0**.0001*
Solitary	61	8		22	
Multiple	43	27		35	
**Differentiation**					
Well/Moderately differentiated	79	21	**0**.0010*	39	**0**.0015*
Poorly differentiated	25	14		18	
**Primary Tumor** (**pT**)			**0**.0003*		0.0017*
pT1	42	6		16	
pT2	32	13		20	
pT3–4	30	16		21	
**AJCC Stage**			**<0**.0001*		**0**.0003*
Stage I	40	4		14	
Stage II	32	13		20	
Stage III-V	32	18		23	
**CLIP score**			**0**.0008*		**0**.0001*
0–1	63	15		29	
2–3	30	16		22	
**Okuda stage**			**0**.0005*		0.0748
I	62	14		31	
II-III	31	17		20	
**IL-20**			**<0**.0001*		**<0**.0001*
Low expression (<5%)	52	8		19	
High expression (≧5%)	52	27		38	

AFP: alpha fetal protein OS: overall survival.

LRFS: local recurrence free survival.

CLIP: The Cancer of the Liver Italian Program.

AJCC: The American Joint Committee on Cancer TNM system.

**Table 3:** Multivariate survival analyses.

Parameter	Category	OS			LRFS		
		RR	95% CI	p-value	RR	95% CI	p-value
**IL-20**	Low expression	1	—	**0**.030*	1	—	**0**.011*
	High expression	3.025	1.110–8.241		2.348	1.211–4.553	
**Pugh-Child’s Classification**	A	1	—	**0**.003*	1	—	0.204
	B	6.048	0.700–52.259		1.546	0.281–8.492	
	C	22.748	2.785–185.791		3.581	0.830–15.451	
**CLIP score**	0–1	1	—	0.939	1	—	**0**.012*
	2–3	1.046	0.324–3.379		3.159	1.293–7.719	
**AJCC Stage**	Stage I	1	—	**0**.004*	1	—	**0**.027*
	Stage II	2.678	0.607–11.813		2.667	1.280–5.555	
	Stage III-V	8.788	2.406–33.489		2.082	0.828–5.237	
**Okuda stage**	I	1	—	0.878	—	—	—
	II-III	1.081	0.341–2.506		—	—	—
**Differentiation**	Well/Moderately differentiated	1	—	0.486	1	—	0.623
	Poorly differentiated	1.592	0.170–2.324		1.270	0.304–2.042	

CLIP: The Cancer of the Liver Italian Program.

AJCC: The American Joint Committee on Cancer TNM system.

OS: Overall survival.

LRFS: local recurrence free survival.

RR: relative risk.

### IL-20 expression was highly associated with cyclin D1 expression in HCC tumor tissue

IL-20 mRNA expression in HCC tumor tissue was significantly higher than non-HCC tissue in 26 patients (Fig. 1d). The upregulated IL-20 and cyclin D1 mRNA expression in patient HCC tissues showed that the IL-20 mRNA expression was highly correlated with cyclin D1 expression (R = 0.658, *P* < 0.0010, Fig. 1e). Both IL-20 and cyclin D1 mRNA were expressed in three different human hepatoma cell lines (Fig. 1f).

### Cell migration and the expression of TNF-α and MMP-13 in IL-20-treated ML-1 cells

**Figure 2:**
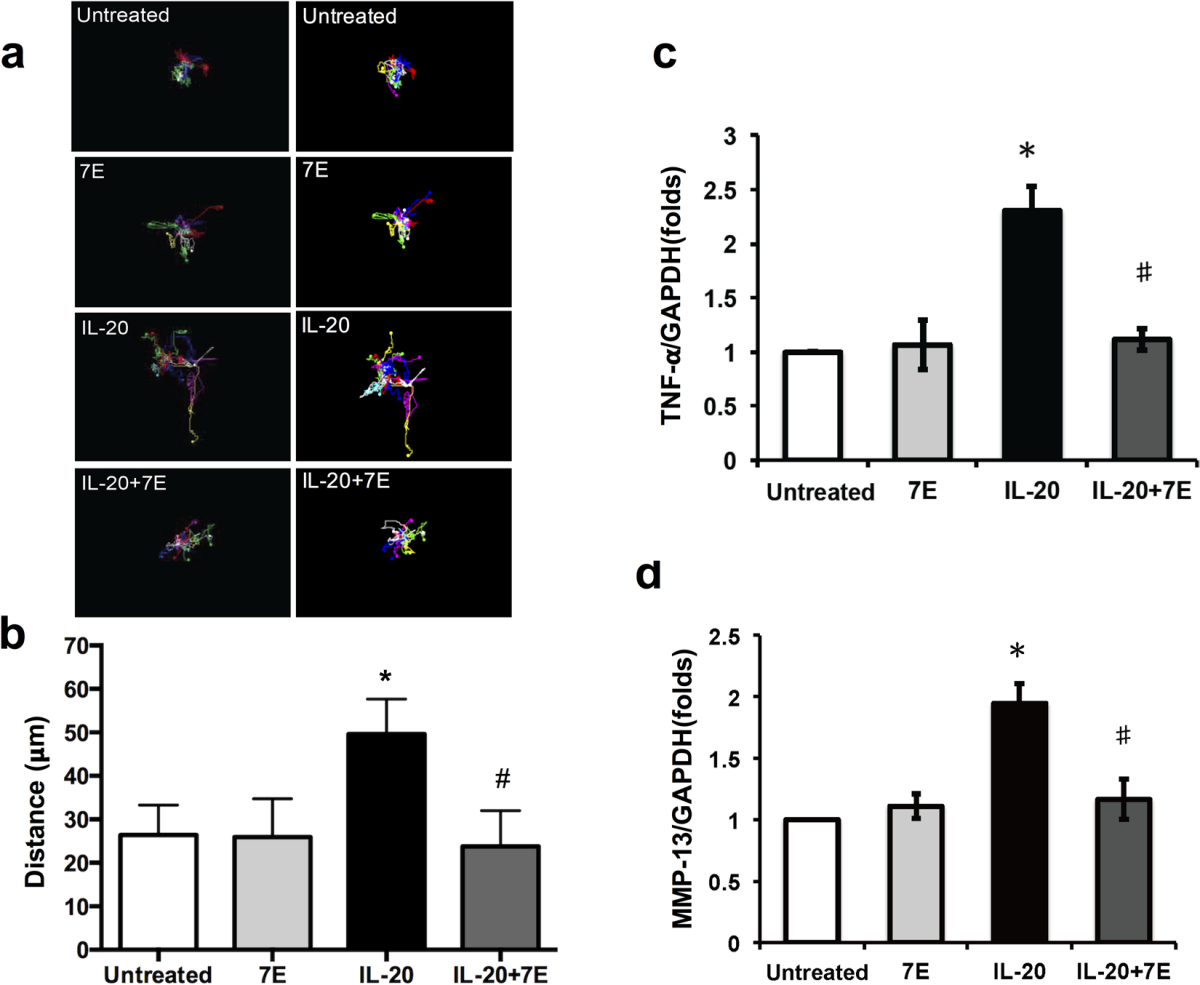
Cell migration, TNF-α expression, and MMP-13 mRNA expression were higher in IL-20-treated ML-1 cells. (**a**,**b**) Cell migration was evaluated using a real time migration assay. ML-1 cells were treated with IL-20 (200 ng/ml), 7E (2 μg/ml), or IL-20 (200 ng/ml) plus 7E (2 μg/ml) for 16 h and monitored. (**a**) Representative time-lapse images for tracking motion distance of ML-1 cells are shown for each group. The motion distance of each cell is presented in different colors (count = 15 cells). (**b**) Quantization of the motion distance (in μm) of ML-1 cells (count = 15 cells). *P < 0.05 compared with the untreated group, ^#^ *P* < 0.05 compared with the IL-20 treated group, each done in quadruplicate. Values are means ± SD. Data are representative of three independent experiments. ML-1 cells were treated with IL-20 (200 ng/ml), 7E (2 μg/ml), or IL-20 (200 ng/ml) plus 7E (2 μg/ml) for 6 h, and the expression levels of TNF-α (**c**) and MMP-13 (**d**) were analyzed using qRT-PCR with specific primers. Quantification analysis of mRNA was normalized with GAPDH. *P < 0.05 compared with the untreated group, ^#^ *P* < 0.05 compared with the IL-20-treated group. Values are means ± SD. Data are representative of three independent experiments.

Immunocytochemical staining and RT-PCR showed that IL-20 and its three receptors—IL-20R1, IL-20R2, and IL-22R1—were all expressed in ML-1 cells and Huh-7 cells (Supplementary Figs S1a and S3a). A Boyden chamber assay (Supplementary Fig. S1b,c) and a real-time migration assay (Fig. 2a,b and Supplementary Fig. S3b) both showed that migration was significantly higher in ML-1 cells and Huh-7 treated with IL-20, the activity was significantly inhibited in those after co-treated with 7E.

We used qRT-PCR to determine the targets directly affected by IL-20; TNF-α, and MMP mRNA expression. We explored the association of IL-20 expression with inflammatory cytokines in HCC tumor formation. TNF-α expression was higher in IL-20-treated ML-1 and Huh-7 cells than in 7E-treated cells (Fig. 2c and Supplementary Fig. S3e). MMP-2, -8, or -12 expression was not significantly changed in ML-1 and Huh-7 cells after IL-20 treatment (data not shown); however MMP-13 expression was significantly upregulated by IL-20, which was inhibited by 7E in ML-1 cells (Fig. 2d). MMP-9 was significantly upregulated by IL-20, which was inhibited by 7E in Huh-7 cells (Supplementary Fig. S3f). These results indicated that IL-20 is an important regulator of tumor progression in HCC.

### Cell proliferation and cyclin D1 expression were inhibited in 7E-treated ML-1 cells

**Figure 3:**
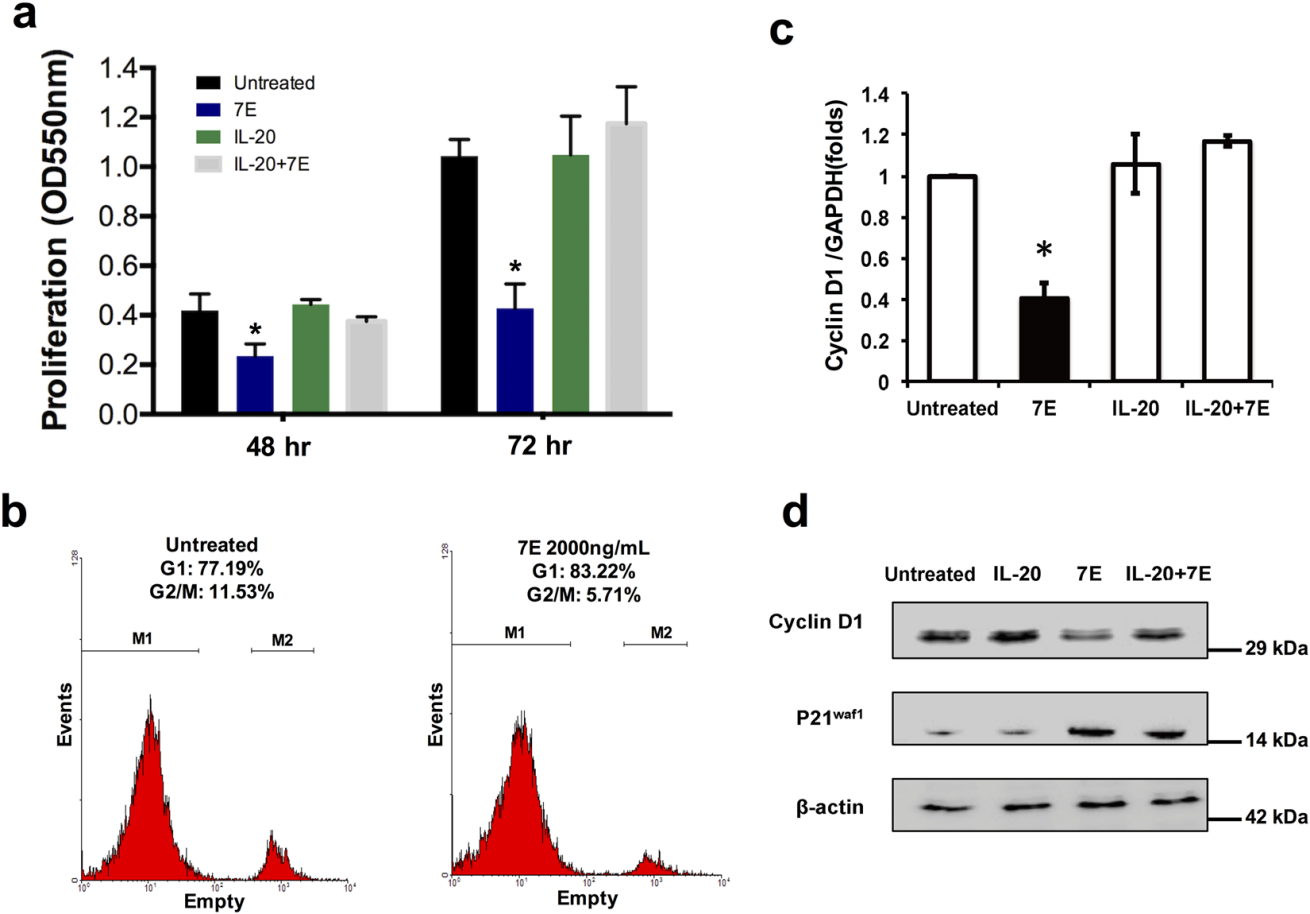
Cell proliferation and cyclin D1 expression was lower in 7E-treated ML-1 cells. (**a**) ML-1 cells were seeded and incubated with IL-20 (200 ng/ml), 7E (2 μg/ml), or IL-20 (200 ng/ml) plus 7E (2 μg/ml) for 48–72 h. Cell proliferation was determined using an MTT assay. Medium alone was used as an untreated control. *P < 0.05 versus the untreated controls. Values are means ± SD. Data are representative of three independent experiments. (**b**) The effects of 7E on cell cycle distribution were measured using flow cytometry. Data are representative of three independent experiments. (**c**) ML-1 cells were treated with IL-20 (200 ng/ml), 7E (2 μg/ml), or IL-20 (200 ng/ml) plus 7E (2 μg/ml) for 6 h, and the expression levels of cyclin D1 were analyzed using qRT-PCR with specific primers. Quantification analysis of mRNA was normalized with GAPDH. *P < 0.05 versus the untreated controls. Values are means ± SD. Data are representative of three independent experiments. (**d**) ML-1 cells were treated with IL-20 (200 ng/ml), 7E (2 μg/ml), or IL-20 (200 ng/ml) plus 7E (2 μg/ml) for 24 h. Cyclin D1 and p21^WAF1^ protein levels were assessed using immunoblotting. β-actin was a loading control. Cropped blots were displayed.

A MTT assay showed that proliferation was inhibited in 7E-treated (2 μg/ml) ML-1 cells (Fig. 3a). Flow cytometry revealed that the percentage of ML-1 cells in the G1 phase was 6% higher after 7E treatment (2 μg/ml); this increase was associated with a concomitant decrease of cells in the G2-M phases of the cell cycle (Fig. 3b).

Because of the G1 arrest in the cell cycle of 7E-treated ML-1 cells, we used qRT-PCR (Fig. 3c) and immunoblotting (Fig. 3d) to analyze the levels of cyclin D1 in those cells. The transcription and protein levels of cyclin D1 were lower, and the p21^WAF1^ protein level was higher in 7E-treated ML-1 cells.

Furthermore, we examined if higher dose of IL-20 regulates the proliferation in ML-1 and Huh-7 cells. A MTT assay showed that the proliferation of ML-1 and Huh-7 cells were higher after the cells had been treated with 400 ng/ml and 1000 ng/ml of IL-20, and that proliferation was inhibited in cells that had been treated with 7E (Supplementary Figs S2a, S3c). Cyclin D1 mRNA expression in ML-1 and Huh-7 cells were also higher after the cells had been treated with 400 ng/ml and 1000 ng/ml of IL-20, and inhibited in cells that had been treated with 7E (Supplementary Figs S2b and S3d).

### Tumor growth *in vivo* was lower in 7E-treated mice

**Figure 4:**
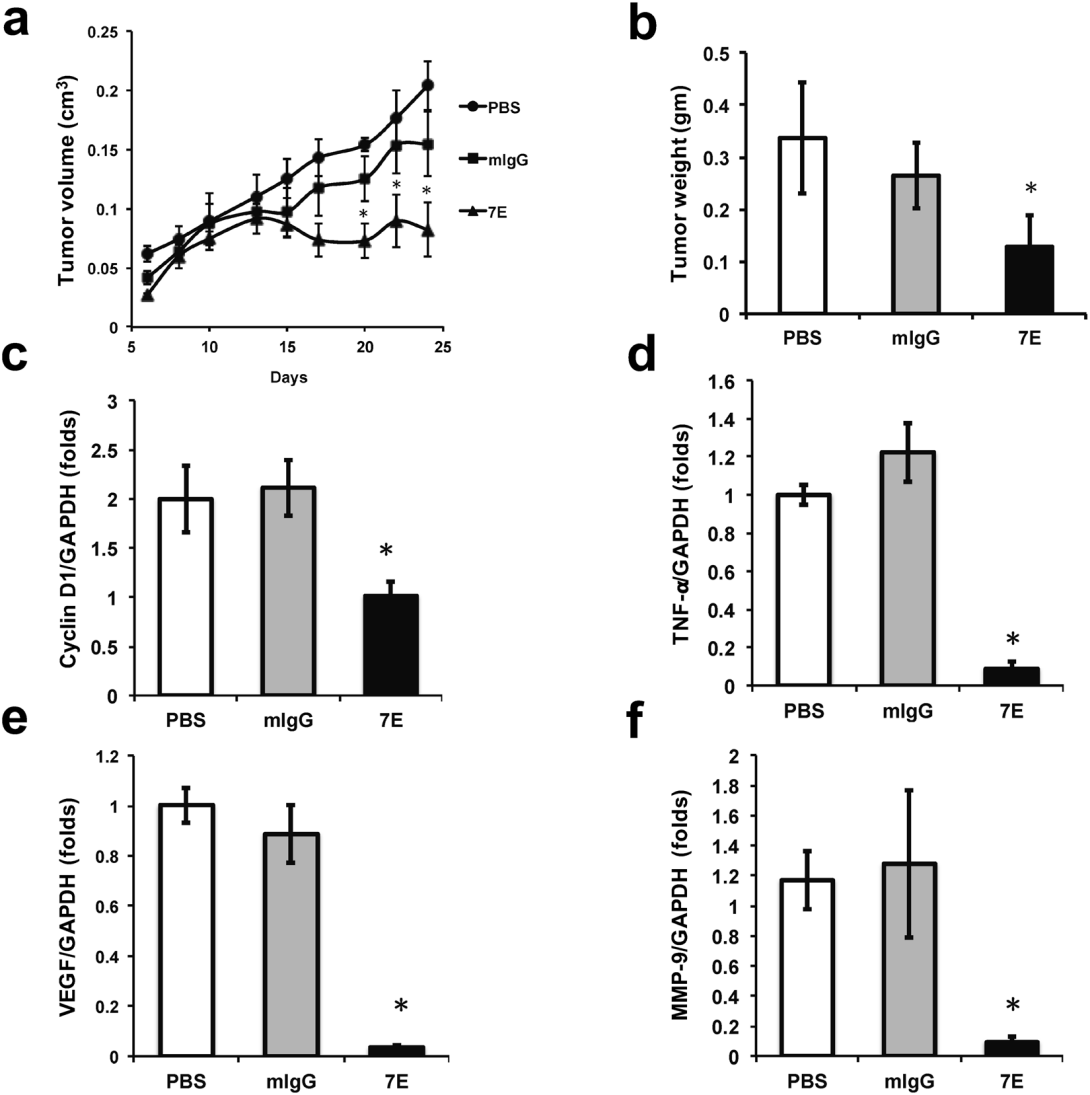
Tumor growth *in vivo* was significantly lower in 7E-treated mice. ML-1 cells were injected into the fat pads of BALB/c mice. One day later, the mice were injected s.c. with PBS (n = 4), 7E (6 mg/kg) and mIgG (6 mg/kg) (n = 5 in each group) twice per week for 25 d. (**A**) Tumor size was measured using a vernier caliper. Values are means ± SD. Data are representative of three independent experiments. (**B**) Mice were killed 25 days after they had been treated with antibody, and their tumors were collected and weighed. Values are means ± SD. Data are representative of three independent experiments. (**C**–**F**) Cyclin D1, TNF-α, VEGF and MMP-9 mRNA expression in tumor tissue was analyzed using qRT-PCR with specific primers. *P < 0.05 versus mIgG controls. Values are means ± SD. Data are representative of three independent experiments.

IL-20 was highly expressed in human HCC tumor tissue, and cell migration was significantly higher in ML-1 cells treated with IL-20. Because migration and proliferation are inhibited in cells treated with 7E, we analyzed tumor growth *in vivo* in BALB/c mice. ML-1 cells were injected into the left fat pads of the mice. They were then injected (s.c.) with PBS, mIgG, or 7E twice per week for the next 25 days. Tumors were smaller and weight was lower in the 7E-treated group than in the mIgG- and PBS-treated groups (Fig. 4A,B and Supplementary Fig. S4). qRT-PCR showed that cyclin D1, TNF-α, VEGF, and MMP-9 mRNA expression were significantly lower in the 7E-treated group than in the mIgG-treated group (Fig. 4C–F). The results demonstrated that IL-20 is involved in the pathogenesis of HCC and 7E significantly inhibited tumor growth.

## Discussion

In this study, we found that IL-20 was critical in HCC. In clinical specimens, IL-20 was highly expressed in HCC tumor tissue. IL-20 expression in HCC patients was correlated with liver cirrhosis, tumor multiplicity and differentiation, and tumor stages. High IL-20 expression in HCC also correlated with poor overall survival (RR > 3). Moreover, IL-20 was positively correlated with cyclin D1 expression in patients with HCC and in human HCC cell lines. In *in vitro* assays, IL-20 increased cell proliferation and migration, and it upregulated cyclin D1, MMP-13, and TNF-α expression in the mouse ML-1 HCC cell line. In *in vivo* assays, the anti-IL-20 mAb 7E attenuated tumor growth in mice injected with ML-1 cells. These indicate that IL-20 is important in the progression of HCC.

Chronic inflammation is frequently linked to the development of cancer. The inflammatory cells and cytokines found in tumors are likely to contribute to tumor growth, progression, and immunosuppression^12^. Multiple risk factors, such as HBV and HCV infections and alcohol-induced chronic inflammation are involved in the development of HCC^13^. Blockading cytokines and chemokines and treatment with nonsteroidal anti-inflammatory drugs reduces the incidence of tumors^12^. Many inflammatory cytokines, such as TNF-α, IL-1, and IL-6 are associated with HCC^3,14,15^. The role of IL-20, as an inflammatory cytokine, in HCC has never been investigated. In this study, IL-20 was highly expressed in HCC tumor tissue and correlated with tumor stages and overall survival. Therefore, IL-20 is important in the pathogenesis of HCC.

Cyclin D1 is well known as an important regulator of cell proliferation and enhancer of several processes relevant to malignant cell transformation, including abnormal growth, angiogenesis, and resistance to apoptosis^16^. Cyclin D1 is overexpressed in human HCC^17^; thus, it is important in human HCC^5^. We found that IL-20 was associated with cyclin D1 mRNA expression in patients with HCC and three human HCC cell lines, which indicates that IL-20 was involved in tumor growth in HCC. Both the proliferation assay *in vitro* and tumor growth assay *in vivo* showed that 7E inhibited cyclin D1-mediated tumor growth, which indicates that IL-20 has an important regulatory role in HCC progression through cyclin D1. The highly correlated expression between IL-20 and cyclin D1 also suggests that IL-20 might be a predictive marker of HCC tumor growth.

We also found that inhibiting tumor cell proliferation using 7E was associated with the arrest of the cell cycle in the G0/G1 phases *in vitro*. Within 24 h after 7E had been added to the cell culture, cyclin D1 protein expression significantly fell and cell cycle inhibitor protein p21^WAF1^ expression significantly rose. The increased p21^WAF1^ expression inhibited cyclin D1/CDK4 complex activity, which explains cell cycle arrest in the G1 phase, and the cell cycle arrest contributed to the 7E-induced inhibition of tumor growth.

However, there were no significant changes in cell proliferation and cyclin D1 expression in ML-1 cells treated with IL-20 (200 ng/ml). This might be attributable to endogenous IL-20 expression in ML-1 cells. Therefore, we used a higher dose of IL-20 (1000 ng/ml). We found that the higher dose of IL-20 (1000 ng/ml) increased cell proliferation. In addition, the higher dose of IL-20 (400 ng/ml and 1000 ng/ml) increased cyclin D1 mRNA expression. Because ML-1 cells already endogenously expressed a high level of IL-20, a much higher dose of exogenous IL-20 was required to observe its effect on cell proliferation.

TNF-α is a major mediator of inflammation. It may act as a tumor promoter by contributing to the tissue remodeling and stromal development necessary for tumor growth and metastasis^18^. TNF-α can induce protease production and vascularization for invasion in tumor cells^12^. We found that IL-20 expression was highly associated with HCC tumor stages in our clinical specimens. IL-20 regulated cell migration and upregulated TNF-α and MMP-13 expression in ML-1 cells. In the mouse model for tumor growth, 7E inhibited both TNF-α expression and tumor growth. Moreover, 7E inhibited MMP-9 and VEGF expression in tumors. This is consistent with our previous finding that IL-20 directly and indirectly induces angiogenesis by regulating VEGF^19^. The matrix metalloproteinases (MMPs) have been shown to be critical modulators of ECM composition and are thus crucial in neoplastic cell invasion and metastasis. Our results demonstrated that IL-20 is involved in many phases of tumor progression: tumor proliferation, migration, and angiogenesis. In addition, IL-20 provides a favorable microenvironment by upregulating proinflammatory cytokines. Therefore, IL-20 produced by cancer cells promotes tumor progression not only by its direct autocrine effect, but also by nurturing a microenvironment for tumor growth and metastasis. 7E inhibited all of these activities *in vitro* and *in vivo* and potently reduced tumor development in HCC. Therefore, we conclude that IL-20 is pivotal in HCC tumor progression. IL-20 might be a useful predictive marker for HCC progression and anti-IL-20 monoclonal antibody 7E has therapeutic potential in HCC.

## Methods

### Clinical specimens

This retrospective study was approved by the Chi-Mei Medical Center Institutional Review Board (IRB No: 10210–003). Signed informed consent was obtained from all participants. The methods were carried out in accordance with the approved guidelines. Blood samples were collected. Tissue samples were obtained from the Chi-Mei Medical Center Tumor and Serum Bank (Tainan, Taiwan). Tumor tissue samples were from 104 patients diagnosed with primary HCC from January 1999 through December 2004. The clinicopathologic variables evaluated are listed in Table 1. For the Cancer of the Liver Italian Program (CLIP) score and Okuda stage analysis, clinical variables obtained from 93 of the 104 patients were analyzed.

Frozen tumor tissue samples from 26 patients with HCC were obtained from the National Cheng Kung University Hospital (Tainan, Taiwan) for quantitative real-time polymerase chain reaction (qRT-PCR) analysis. The study was approved by the Ethics Committee of National Cheng Kung University Hospital (IRB No: B-ER-104-393). Signed informed consent was obtained from all participants. The methods were carried out in accordance with the approved guidelines. Blood samples were collected. Patient demographic and clinical information is listed in Supplementary Table 1.

### Expression and purification of human and mouse IL-20 recombinant protein

The human IL-20 fragment from Leu^25^ to Glu^176^ and mouse IL-20 fragment from Leu^25^ to Leu^176^ was amplified using PCR and inserted into the mammalian expression vector, pSecTag2A (Invitrogen, Carlsbad, Calif.). A Tag consisting of six histidine residues was placed at the C-terminus of the recombinant proteins. The protein was expressed in 293T cells and purified using metal affinity chromatography, as previously described^19,20^. The purity of the eluted fractions from the affinity column was checked by the SDS-PAGE test in a reducing condition according to the standard protocol. The purity of human and mouse IL-20 proteins was about 95%.

### Reverse transcription (RT-PCR)

The total RNA of cells and tumor tissue was extracted using Trizol reagent (Invitrogen, Carlsbad, CA) and underwent reverse transcription according to the manufacturer’s instructions. The expression of mRNA was analyzed using amplified PCR and real-time PCR with gene-specific primers. GAPDH was as an internal control.

### Quantitative real-time polymerase chain reaction (qRT-PCR)

Total RNA was isolated. Reverse transcription (RT) was done with reverse transcriptase (Thermo Scientific, Rockford, IL). The mRNA expression was then amplified on a thermocycler (LightCycler 480; Roche Diagnostics, Indianapolis, IN), with SYBR Green I (Roche Diagnostics) as the interaction agent. The quantitative analysis of mRNA was normalized with GAPDH as the housekeeping gene. Relative multiples of changes in mRNA expression were determined by calculating 2^−ΔΔCt^. Primer sequence is listed in Supplementary Table 2.

### Immunohistochemistry staining

Paraffin-embedded-tissue samples were used for immunohistochemistry staining with purified 7E (2 μg/ml) at 4 °C overnight^21,22^. Incubating paraffin tissue sections with mouse IgG isotype (R&D Systems, Minneapolis, MN) instead of primary antibody was the negative control. Two investigators trained in liver pathology and blinded to the sample sources analyzed the histology and the IL-20 expression levels of at least five sections from each patient. The scoring of immunohistochemistry stains in each specimen was determined using a histological score (H)^23^ that was calculated using the following equation: H = Σ*Pi* (*i* + 1), where *i* is the staining intensity of the stained tumor cells (0–3+), and *Pi* is the percentage (range: 0–100%) of stained tumor cells for each intensity. The IL-20 immunostaining was labeled low-grade (H < medium) or high-grade (H ≥ medium).

### Cell culture

The murine HCC cell line ML-1 and human HCC cell line Huh-7 was purchased from The Food Industry Research and Development Institute (Hsinchu, Taiwan). Cells were grown in Dulbecco’s modified Eagle’s minimal essential medium (DMEM) (Hyclone, Logan, UT) supplemented with 10% (v/v) FBS (Hyclone), 100 U/ml of penicillin, and 100 mg/ml of streptomycin (Hyclone) and kept at 37 °C in a 5% CO2/95% air atmosphere.

### Immunocytochemical staining

ML-1 and Huh-7 cells were fixed in 3.7% paraformaldehyde and then permeabilized using PBS with 0.1% Triton X-100. The cells were blocked by immersing them in antibody diluent with background reducing components (Dakocytomation, Carpinteria, CA) and then incubated with primary antibody in blocking reagent. Immunocytochemical analysis was done using a chromogen kit (Romulin AEC Chromogen Kit; Biocare Medical, Walnut Creek, CA) and counterstained with Mayer’s hematoxylin (J. T. Baker, Phillipsburg, NJ). The antibodies used were IL-20R1, IL-20R2, IL-22R1(R&D system), and mIgG isotype controls.

### Migration assays

The migration assay was done using a Boyden chamber housing a polycarbonate filter with 8-μm pores (Nucleopore, Cabin John, MD). The upper wells were loaded with 10^4^ ML-1 cells. The lower chambers were filled with conditioned medium. The cells were incubated for 16 h at 37 °C. Cells adhering to the lower side of the filter were fixed in 100% methanol and stained with Liu’s staining (Tonyar Biotech, Tao-Yuan, Taiwan) for counting. The number of cells on the lower surface of the filter was determined microscopically by counting 3 randomly selected fields.

The migration assay was also done using a real-time migration assay. ML-1 and Huh-7 cells were seeded at 5 × 10^4^ cells/per well in 3.5-cm dishes and allowed to attach for 24 h. The cells were then exposed to conditioned medium. Cell migration kinetics was recorded (JuLI Smart Fluorescent Cell Analyser; Montreal Biotech) for approximately 16 h. The result was then analyzed using ImageJ Software (http://imagej.nih.gov/ij/download.html).

### Cell proliferation assay

ML-1 cells were plated at a density of 4000 cells/ in 48-well plate and incubated with IL-20 (200 ng/ml), 7E (2 μg/ml), or IL-20 (200 ng/ml) plus 7E (2 μg/ml) for 48–72 h. Cell proliferation was determined using an MTT (3-[4,5-dimethylthiazol-2-yl]-2,5- diphenyltetrazolium bromide) assay (Sigma-Aldrich, St. Louis, MO). After the cells had been incubated in conditioned medium for 48–72 h, 0.5 mg/mL of MTT was added to each well and incubated for 3 h. The supernatant was aspirated, and DMSO (Sigma-Aldrich) was added to dissolve the blue crystals. Absorbance was determined at 550 nm.

### Flow cytometry

ML-1 cells were plated at a density of 10^5^ cells/ml in 6-well plate. After they had been incubated with conditioned medium for 24 h, the cells were harvested by trypsinization, collected by centrifugation, and fixed with 70% ethanol overnight. After they had been washed three times with cold PBS, the cells were stained with 100 μg/ml of propidium iodide (PI) and 50 μg/ml of DNase-free RNase A (Sigma-Aldrich) for 10 min. A flow cytometer (FACScan; Becton Dickinson, Franklin Lakes, NJ) was used to evaluate their DNA fluorescence. The percentages of cells in the sub-G_0_/G_1_ and G_2_/M phases were quantified using WinMDI 2.8 software.

### Immunoblotting

Cells were stimulated with conditioned medium for 24 h. Immunoblotting used antibody specific for p21 (GeneTex International, Hsinchu, Taiwan) and Cyclin D1 (Cell Signaling, Beverly, MA). β-actin (GeneTex) was used as a loading control.

### Tumor growth mouse model

All animal experiments were done according to the protocols of the National Institutes of Health standards and guidelines for the care and use of experimental animals. The research procedures were approved by the Animal Ethics Committee of National Cheng Kung University (IACUC approval No: 105087). The methods were carried out in accordance with the approved guidelines. All efforts were made to minimize animal suffering and to reduce the number of animals used. Male 8-week-old BALB/c mice were used in all experiments. For the tumor growth mouse model, the left fat pad of each mouse was injected s.c. with 10^6^ ML-1 cells. The mice were then randomly assigned to one of three groups (n = 5 in each group) and treated with PBS, 7E (6 mg/kg; s.c.), or mouse IgG (6 mg/kg; s.c.) twice per week for the duration of the experiment. Twenty-five days after the tumor cells had been injected, the mice were killed and the tumor tissue was harvested.

### Follow-up and statistical analysis

Statistical analysis was done using SPSS 14.0 (SPSS Inc., Chicago, IL). Associations and comparisons of IL-20 expression with various parameters were evaluated using Student’s *t* tests or Spearman correlation analysis. In this cohort, 104 patients had available follow-up data in August 2012 (median: 35.3 mo; mean: 40.1 mo; range: 1.0–100.7 mo). The endpoint analyzed was the overall survival (OS) or local recurrence-free survival (LRFS) of patients with low- and high-grade IL-20 expression, assessed using Kaplan-Meier methods and compared using a log-rank test. The correlation between IL-20 expression and various clinicopathologic examinations was evaluated using a χ^2^ test. Other results are means ± SD. Significance was set at *P* < 0.05.

## Electronic supplementary material

Supplementary information
