# Supplementary material for: Anti-IL-20 monoclonal antibody inhibited tumor growth in hepatocellular carcinoma
Source: Sci Rep. 2017 Dec 14;7:17609. doi: 10.1038/s41598-017-17054-1 (PMC5730582; doi:10.1038/s41598-017-17054-1)
Supplement: Supplementary file 1 — Supplementary information [file 41598_2017_17054_MOESM1_ESM.pdf]

**Anti-IL-20 monoclonal antibody inhibited tumor growth in hepatocellular carcinoma**

Yi-Shu Chiu[1], Chung-Hsi Hsing[3*], Chien-Feng Li[4,5,6*], Chon-Yee Lee[2], Yu-Hsiang Hsu[7,8] and Ming-Shi Chang[1,2]

[1]Institute of Biopharmaceutical Sciences, [2]Department of Biochemistry and Molecular Biology and [7]Institute of Clinical Medicine, College of Medicine, National Cheng Kung University, Tainan, Taiwan

[3]Department of Anesthesiology and [4]Department of Pathology, Chi-Mei Medical Center, Tainan, Taiwan

[5]National Institute of Cancer Research, National Health Research Institutes, Tainan, Taiwan

[6]Department of Biotechnology, Southern Taiwan University of Science and Technology, Tainan, Taiwan

[8]Research Center of Clinical Medicine, National Cheng Kung University Hospital, College of Medicine, National Cheng Kung University, Tainan, Taiwan

*Equal contribution to these authors

**Supplementary Table**

**Supplementary Table S1. Demographic and clinical information**

| Variable | Value |
|---|---|
| Age (years) | 46-80 |
| Males:Females | 15:11 |
| HBV(–), HCV(–) | 5 |
| HBV(+), HCV(–) | 12 |
| HCV(+), HBV(–) | 8 |
| HBV(+), HCV(+) | 1 |

HBV, hepatitis B virus; HCV, hepatitis C virus; (+), positive; (–), negative.

**Supplementary Table S2. Primer sequences**

| Gene | Forward (5' to 3') | Reverse (5' to 3') |
|---|---|---|
| **hIL-20** | AAGACACAAAGCCTGCGAAT | TGGAGCTCTTGACCTCGAAT |
| **hCyclin D1** | CACACGGACTACAGGGGAGT | AGGAAGCGGTCCAGGTAGTT |
| **hTNF-α** | AGCCCATGTTGTAGCAAACC | GGTTGAGGGTGTCTGAAGGA |
| **hMMP-9** | GATGCGTGGAGAGTCGAAAT | GATGCGTGGAGAGTCGAAAT |
| **hMMP-13** | GAGCACCCTTCTCATGACCTC | GGCATTCCACCACTGCTCCC |
| **hVEGF** | GTGTGCGCAGACAGTGCT | CTGCATGGTGATGTTGGACT |
| **hGAPDH** | GTATCGTGGAAGGACTCATG | TCTTCCTCTTGTGCTCTTGC |
| **mIL-20** | GAGATTCGGGATAGTGTGCA | CTGCCTGAAGTTCCAACTCT |
| **mCyclin D1** | GAGGAGCTGCTGCAAATGG | TGGAGGGTGGGTTGGAAAT |
| **mTNF-α** | AGTGACAAGCCCGTAGCCC | AGCCTTGTCCCTTGAAGAG |
| **mMMP-9** | CGTCGTGATCCCCACTTACT | AGA GTA CTGCTTGCCCAGGA |
| **mMMP-13** | CCTGGACCAAACTATGGTGGG | AAGCTCATGGGCAGCAACAA |
| **mVEGF** | CACAGCAGATGTGAATGCAG | TTTACACGTCTGCGGATCTT |
| **mGAPDH** | AGCCTCGTCCCGTAGACA | GATGACAAGCTTCCCATC |

## Supplementary Figure

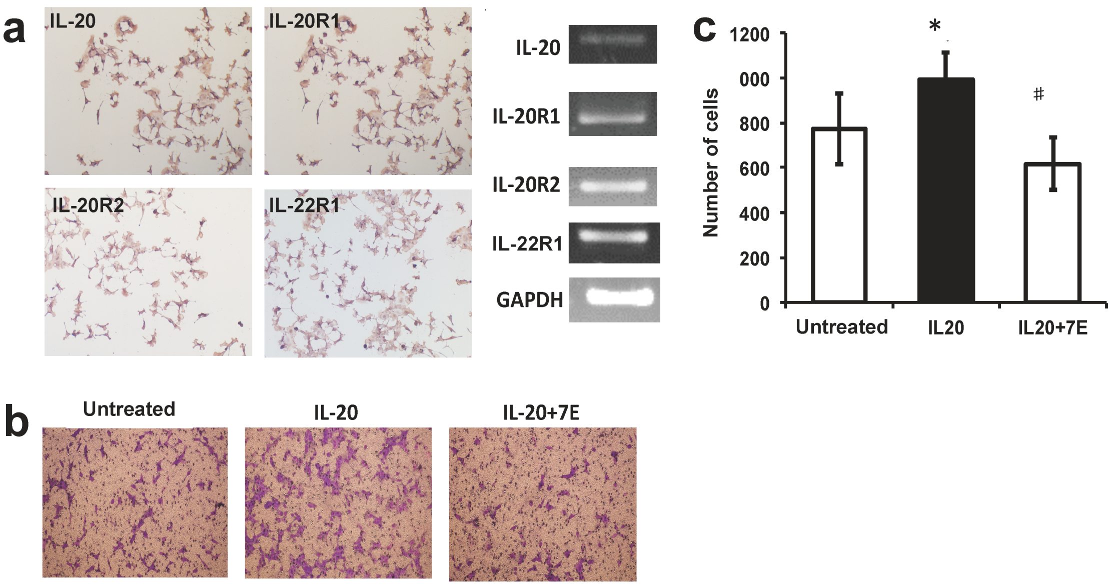


**Supplementary Fig. S1. Cell migration was higher in IL-20-treated ML-1 cells.**

(a) ICC staining and RT-PCR showed that IL-20 and its receptors were expressed in ML-1 cells. Cell migration was evaluated using a Boyden chamber assay. ML-1 cells were treated with IL-20 (200 ng/ml), or IL-20 (200 ng/ml) plus 7E (2 µg/ml) for 16 h. Representative Liu's stain photomicrographs (b) and the number of cells (c) are shown for each group. Untreated ML-1 cells in medium alone were used as controls. *P < 0.05 versus untreated controls. #P < 0.05 compared with the IL-20-treated group. Values are means ± SD. Data are representative of three independent experiments.

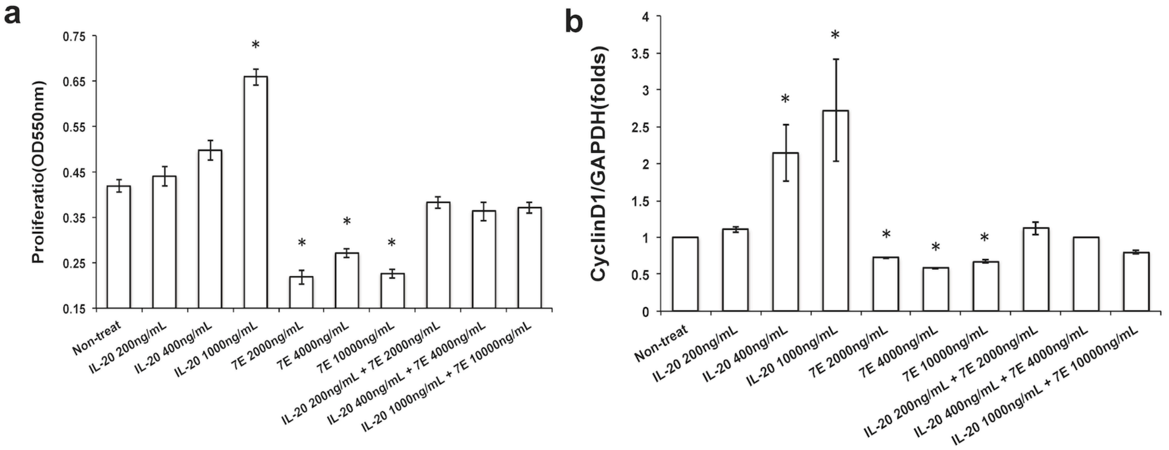


**Supplementary Fig. S2. Cell proliferation and cyclin D1 mRNA expression were higher in ML-1 cells treated with a high dose of IL-20.** (a) ML-1 cells were seeded and incubated with conditioned medium for 72 h. Cell proliferation was determined using an MTT assay. Untreated ML-1 cells in medium alone were used as controls. *P < 0.05 versus untreated controls. Values are means ± SD. Data are representative of three independent experiments. (b) ML-1 cells were treated with conditioned medium for 6 h, and the expression level of cyclin D1 was analyzed using qRT-PCR with specific primers. Quantification analysis of mRNA was normalized with GAPDH. *P < 0.05 versus untreated controls. Values are means ± SD. Data are representative of three independent experiments.

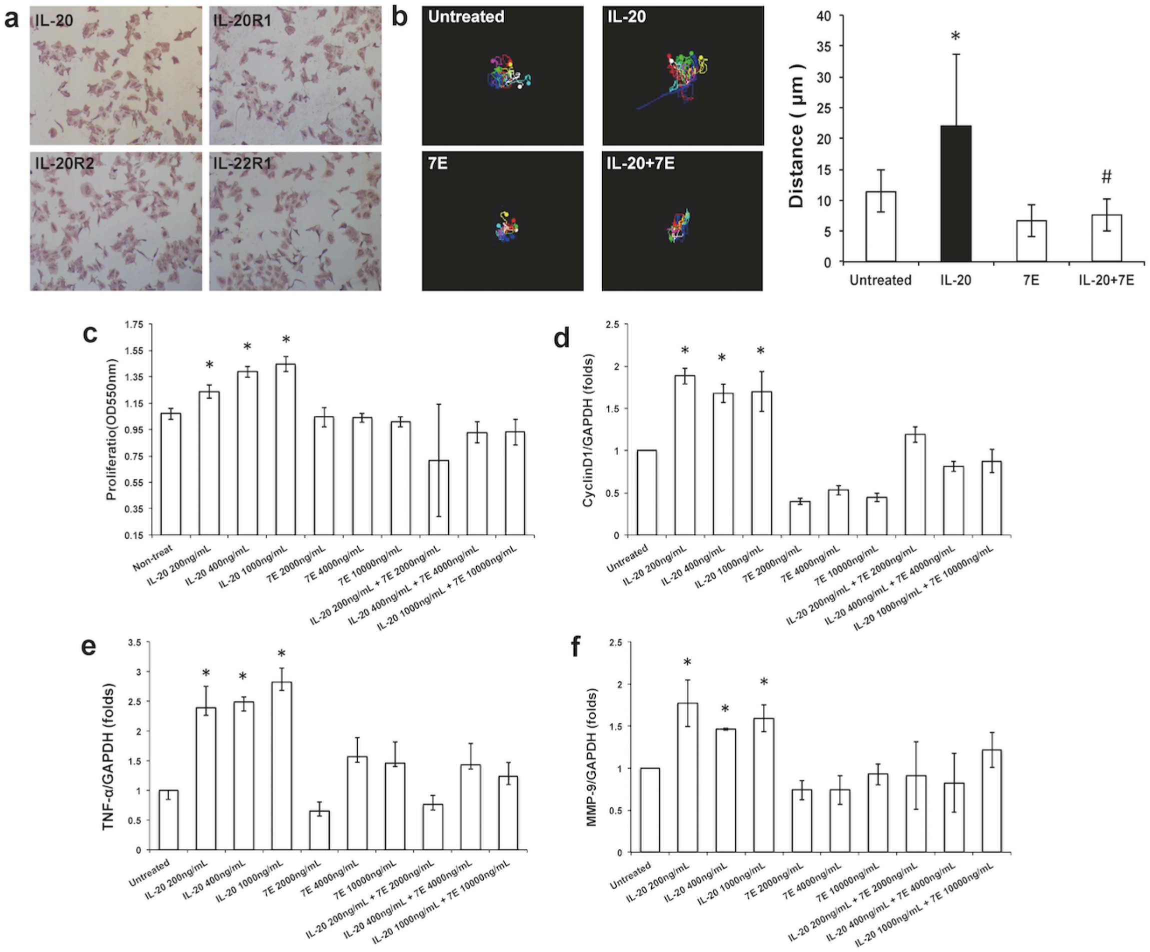


**Supplementary Fig. S3. Cell migration and proliferation were higher in IL-20-treated Huh-7 cells.** (a) ICC showed that IL-20 and its receptors were expressed in Huh-7 cells. (b) Cell migration was evaluated using a real time migration assay. Huh-7 cells were treated with IL-20 (200 ng/ml), 7E (2 μg/ml), or IL-20 (200 ng/ml) plus 7E (2 μg/ml) for 16 h and monitored. Quantification of the motion distance (in μm) of Huh-7 cells (count = 10 cells). (c) Huh-7 cells were seeded and incubated with conditioned medium for 72 h. Cell proliferation was determined using an MTT assay. Huh-7 cells were treated with conditioned medium for 6 h, and the expression level of cyclin D1 (d), TNF-α (e) and MMP-9 (f) were analyzed using qRT-PCR with specific primers. Quantification analysis of mRNA was normalized with GAPDH. Untreated Huh-7 cells in medium alone were used as controls. *P < 0.05 versus untreated controls. Values are means ± SD. Data are representative of three independent experiments.

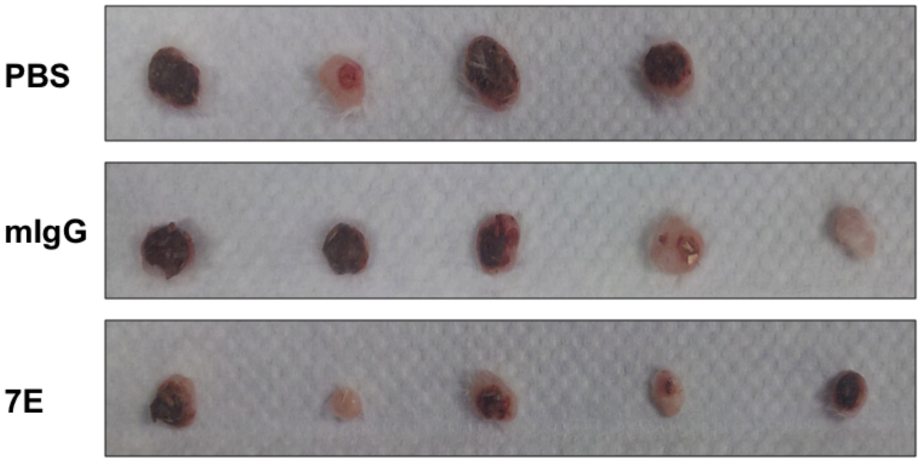

**Supplementary Fig. S4. Tumor growth *in vivo* was lower in 7E-treated mice.** ML-1 cells were injected into the fat pads of BALB/c mice. One day later, the mice were injected s.c. with PBS (n = 4), 7E (6 mg/kg) and mIgG (6 mg/kg) (n = 5 in each group) twice per week for 25 d. Mice were killed 25 days after they had been treated with antibody, and their tumors were collected and weighed. Values are means ± SD. Data are representative of three independent experiments.
